# INFLUENCE OF HEALTH AND SOCIAL SUPPORTS ON OUTCOMES POST-REHABILITATION FOR HIP FRACTURE SURGERY: SYSTEMATIC REVIEW

**DOI:** 10.1093/geroni/igz038.2702

**Published:** 2019-11-08

**Authors:** Katherine McGilton, Shirin Vellani, Melanie Bayly, Elizabeth Tanjong-Ghogomu, Andrea Iaboni, Julie Lapenskie, Vivian Welch, Abeer Omar

**Affiliations:** 1 KITE, Toronto Rehab, University Health Network, Toronto, Ontario, Canada; 2 Canadian Centre for Health and Safety in Agriculture (CCHSA), University of Saskatchewan, Saskatoon, Saskatchewan, Canada; 3 Bruyère Research Institute, University of Ottawa, Ottawa, Ontario, Canada; 4 University Health Network, Toronto, Ontario, Canada; 5 Bruyere Research Institute | Institut de recherche Bruyere Ottawa Hospital Research Institute | Institut de recherche de l’Hôpital d'Ottawa, Ottawa, Ontario, Canada; 6 Centre for Global Health, Bruyère Research Institute, Ottawa, Ontario, Canada; 7 KITE, Toronto Rehab, University Health network, Toronto, Ontario, Canada

## Abstract

Background: Older adults who sustain hip fractures encounter physical and functional decline after discharge from inpatient rehabilitation. Currently, a synthesis of literature is lacking on health and social supports that may impact outcomes in the community-dwelling older adults, post-discharge from rehabilitation. Methodology: We conducted a systematic review to a) evaluate how health and social supports influence outcomes for older adults and their caregivers following inpatient rehabilitation post-hip fracture surgery, and b) identify the factors that affect their impact on outcomes. We searched Medline, CINAHL, Embase, Emcare, Psychinfo, and Ageline for publications between 2000 and 2018. We followed Cochrane Handbook methods to screen titles and abstracts, appraise quality, collect data and synthesize results. Results: A total of 3364 articles were retrieved, and 34 studies were included for final synthesis, including 24 randomized control trials and 10 observational studies. Most studies excluded persons with moderate or severe cognitive impairment. Interventions can be broadly categorized as either comprehensive care delivered by interdisciplinary teams focusing on exercise, nutrition and fracture prevention; or exercise sessions delivered by health professionals, trained instructors or volunteers. Interventions involving interdisciplinary teams demonstrated moderate improvement of mobility and functional ability in the first 3 months. However, the longitudinal effects of interventions were not realized for all. Conclusion: This review provides evidence of the effectiveness of health and social supports provided to older adults post-hip fracture. We are uncertain of the applicability to people with cognitive decline due to exclusion from most studies. Implications for practice and research will be discussed.

